# Macroheterogeneities Induced by Disulfide Bond Reduction in Native Mucus

**DOI:** 10.1021/acsabm.4c01758

**Published:** 2025-05-19

**Authors:** Giorgia Franzino, Fabiana Tescione, Domenico Larobina

**Affiliations:** Institute of Polymers, Composites and Biomaterials, 9327 National Research Council of Italy, Piazzale E. Fermi 1, 80055 Portici, Naples, Italy

**Keywords:** macroheterogeneity, gastric mucus, disulfide reduction, photon correlation imaging, rheology

## Abstract

Disulfide bond reducing agents have long been used as therapeutic drugs (mucolytics) for mucus hypersecretions. Breakage of disulfide bridges is known to cause a reduction in the degree of cross-linking, making the mucus more fluid. In addition to the drop in viscoelastic properties, the disulfide breakage is also known to affect the structure of the mucus on both micro- and mesoscale. Despite this knowledge, little is known about the reorganization of the mucus at the macroscopic scale. This contribution explores the effect of the reducing agent, tris­(2-carboxyethyl)­phosphine hydrochloride (TCEP), on the structure of native porcine gastric mucus. After exposing the mucus to increasing concentrations of TCEP, we measure the macroscopic rheological properties along with its microscopic dynamics. The results obtained show an increase in macroscopic heterogeneity with TCEP, which we attribute to the mucus tendency to phase separate. Furthermore, we examine the soluble fractions of the different reduced mucuses by measuring the size of the suspendable macromolecules. The results indicate that the size remains approximately constant with an increasing TCEP concentration. Our contribution may be important to describe the effects of a disulfide reducing agent on the mucus structure and, consequently, on mucociliary and cough clearance.

## Graphical abstract

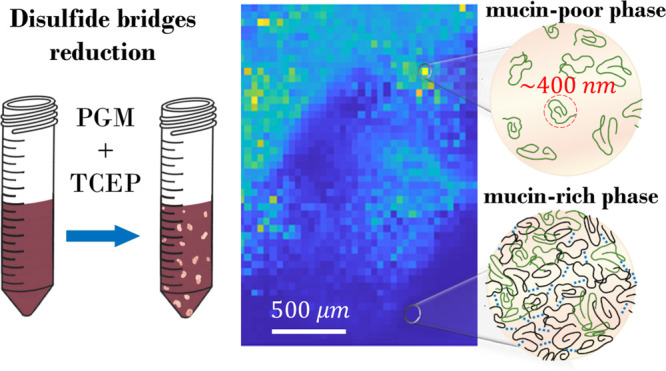

## 1 Introduction

Mucus is a biological fluid that serves various functions in mammals: it keeps the epithelium hydrated, prevents the spread of bacteria and pathogens, and is the basis of the epithelium cleaning mechanism known as mucociliary clearance (MCC).^1–3^

Compositionally, mucus is made up of mainly water, with a total organic solid fraction ranging from a few percent up to over 10%, depending on the epithelium and the possible presence of pathologies. The solid fraction, in turn, is composed of salts, nonmucin proteins, lipids, DNA traces, and 20–30% (0.5–5.0% of the total) of glycoproteins known as mucins.^4,5^ Mucins constitute the main macromolecular component present in mucus and dominate its rheological behavior.^6^ They are identified by the tissue expression (e.g., pulmonary, gastric, etc.) and their location in the mucosa (e.g., membrane-bound vs gel-forming mucins).^7,8^ Although there are compositional variations among the various mucins, their general structures are similar and are characterized by a peptide backbone chain to which sugar oligomers are bonded. The backbone contains cysteine zones, predominantly located toward the ends, forming two types of disulfide bridges: intra- and interchain. The intrachain bridges have the role of stabilizing the conformation of the protein, while the interchain ones, mainly associated with gel-forming mucins, lead to the formation of mucin multimers. Indeed, a single mucin unit, often called macromonomers (because of its high molecular weight ∼ 0.5 MDa with *R*_g_ ≃ 20 nm),^9–11^ can give rise to linear multimers by terminal disulfide bonds. To have an idea of the dimensions of mucin multimers, it must be considered that gel-forming mucins, e.g., MUC5AC, can exceed 20 MDa (i.e., about 50 macromonomers) with a radius of gyration *R*_g_ ≃ 200 nm.^12,13^ In addition to the two types of disulfide bridges mentioned above, we must also include a third one due to cysteines belonging to nonmucin proteins. In this regard, it is worth mentioning the presence inside the mucus of the trefoil factors (TFFs), which have long been indicated to contribute to the structure and rheology of mucus.^14^

To perform its various biological functions, the properties of mucus must fall within a certain physiological range.^15^ Pathological variations observed in muco-obstructive diseases, such as cystic fibrosis or chronic obstructive pulmonary disease, are associated with alteration related to mucus hypersecretion.^16,17^ Indeed, a change in the composition and solid fraction of mucus causes a variation of its physiological behavior, leading, in the case of pulmonary mucus, to failure of the MCC and cough mechanisms.^16^ To counteract these complications, current clinical practice relies on the use, among others, of mucolytic agents (e.g., disulfide bond reducing agent).^3^ Acting at the molecular level, these components reduce the elasticity of the mucus, thereby increasing its fluidity and promoting clearance mechanisms. Putatively, the effect at the molecular level is to reduce the mucin molecular weight, i.e., to cleave intramacromonomeric disulfide bridges.^18–20^ However, due to the large number of disulfide bonds present in mucus, this hypothesis has been recently questioned.^3,21^

The study of healthy and pathological mucus (mainly of lung type), as well as the effect induced by different perturbations, has been the topic of a considerable number of contributions.^21–24^ In this respect, both macroscopic (rheology) and microscopic (particle tracking) measurements have been widely employed to reveal the behavior of mucus.^15,25–28^ Combining observation on different length scales, Wagner et al. analyzed the effect of different perturbations, such as the pH, surfactants, and salt concentration, on a gel of MUC5AC extracted from porcine gastric mucus (PGM).^27^ The authors demonstrated that different perturbations could stiffen the mucin gel by acting on different specific interactions present within the mucins. Although mechanically similar, the mesoscopic structures resulting from these perturbations are different. Specifically, variations in pH induce higher mechanical properties through mesoscopic phase separation, while changes in the ionic strength only cause stronger associations between mucin multimers without altering its microscopic structure. Multiple levels of assembly at different length scales was also described by Meldrum et al. on a gel reconstructed from purified intestinal mucus, under nondenaturing conditions.^21^ In their work, the authors showed the presence of a “hierarchical assembly of mucins and non-mucin proteins”. They depicted mucus as “clusters of mucins, forming a network of microdomain that assembles into a yield stress fluid, exhibiting a thixotropic rheological behavior”. In these contributions, the authors assert that the observed behavior is a consequence of the different interactions that can be formed between mucin multimers. Indeed, the simultaneous presence of negative charges capable of forming salt bridges, disulfide bridges capable of stabilizing protein regions, and hydrophobic groups capable of forming intramucin cross-links^6,29^ gives mucus the ability to reorganize in response to external perturbations. Such a reorganization is connected to conformational variations (unfolding) of mucin macromonomer.^30^ This is the case that occurs when the pH changes. Upon unfolding, in fact, mucins expose hydrophobic sites previously hidden, inducing a reorganization of the mucus structure (phase separation).^27,31^

Despite the extensive description of mucus changes following various perturbations, information about the effect of disulfide bond reduction on mucus reorganization is minimal. Knowledge of this reorganization would allow us to describe the macroscopic and microscopic properties of mucus, especially in pathological mucus, where mucolytic agents are used. A thorough description of the effect of disulfide reducing agents on different length scales could therefore help us to understand phenomena such as mucociliary and cough clearance.

In the following sections, we reported the effect of a reducing agent, tris­(2-carboxyethyl)­phosphine hydrochloride (TCEP), on native PGM. The choice of TCEP as the reducing agent is dictated by its biocompatibility, its ability to be active even at low pH, and its efficacy. The use of PGM is essentially linked to practical reasons, that is, the ease of obtaining the material and the lack of authorization from ethics committees.

## 2 Materials and Methods

### 2.1 Materials

Tris­(2-carboxyethyl)­phosphine (TCEP), sodium azide (NaN_3_), sodium chloride (NaCl), disodium hydrogen phosphate (Na_2_HPO_4_), sodium hydroxide (NaOH), and ethylenediaminetetraacetic acid disodium salt solution (0.1 M EDTA) were all purchased from Sigma-Aldrich and used as received.

### 2.2 Experimental Procedure

Phosphate buffer solution (PBS) was prepared by dissolving 0.05 mol of Na_2_HPO_4_ and 0.003 mol of NaN_3_ in 450 mL of distilled water. We then added 50 mL of 0.1 M EDTA and 0.1 mol of NaCl and finally adjusted the pH to 8 using 1 M NaOH (≈2.5 mL).

PGM was collected from stomachs of just-slaughtered pigs and used without prior purification. Briefly, the stomachs were opened, and the inner wall was washed with deionized water to remove debris. Pieces of approximately 10 × 10 cm^2^ were cut and frozen until use. Prior to each analysis, a piece of stomach was thawed at room temperature, and the mucus was collected by directly scraping from its surface. We then measured the pH (by Hanna Edge HI2002-02 pH-meter) and the dry fraction (by vacuum drying overnight at 50 °C), obtaining values of 6.0 ± 0.5 and 8.0 ± 0.9%, respectively. Finally, we added 0.02% (w/w) NaN_3_ to inhibit bacterial growth.

To evaluate the effect of the reducing agent, samples were added with a fixed volume (0.16 mL/g_PGM_) of a TCEP solution at four different molarities. After mixing, the final concentrations were 0, 0.8, 1.76, or 2.64 μmol_TCEP_/g_PGM_. After TCEP addition, the samples were mixed with a spatula, incubated at 37 °C for 30 min, and finally tested.

### 2.3 Rheology

All rheological tests were conducted at 25 °C with a rotational rheometer (Thermo Scientific HAAKE MARS III) using a 35 mm plate–plate geometry at a gap ranging between 440 and 660 μm, unless otherwise explicitly stated. As an example, we report the gaps used for the startup tests in Table T1. To prevent water evaporation, a solvent trap system was used. After loading of the sample, an equilibrium time of ∼20 min was allowed to relax the normal force to a value comparable to the detectable minimum (0.01 N). This equilibrium time is the result of a compromise between the rate of relaxation of the imposed stress and the rate at which the material ages. In fact, from measurements conducted in the past on the PGM, we know that mucus tends to slowly relax the imposed stress^32^ and to evolve spontaneously over time.^33^

We performed both frequency sweep tests in the linear regime and startup tests in the nonlinear regime. To determine the linear viscoelasticity regime, preliminary amplitude sweep tests at different frequencies (5, 0.5, and 0.05 Hz) were also performed (Figures S2–S4). For the frequency sweep tests, storage (*G*′) and loss (*G*″) moduli were measured in the range of 0.02–20 Hz, by applying a variable stress between 0.1 and 1 Pa, as dictated by the preliminary amplitude sweep tests. However, data at frequency *f*≳ 6 Hz (ω ≳ 37 rad/s) were inaccurate due to the inertia of the instrument. In Figures S5 and S6, we reported the spectrum of the raw phase angle for two representative samples, where it is evident that δ_R_ exceed 90° for *f*≳ 6 Hz . In our analysis, we therefore cut the data above this threshold. For the shear startup experiment, instead, $\mathrm{\gamma ̇}=1\;s^{-1}$ was instantaneously applied to the sample and kept for ∼60 s, while the stress (σ) was recorded.

Due to the biological origin of mucus, the tests performed always display a sample-to-sample variability. For the oscillatory linear tests, such variability is mainly observed in the amplitude of the shear moduli at a fixed TCEP concentration, while the frequency dependencies always appear characteristically the same. To highlight these dependences, we decided, for each test, to normalize both moduli with respect to the value of the storage modulus evaluated at a reference frequency of 1 Hz:

1 $${\mathrm{G̃}}^{\prime }\equiv G^{\prime }/G^{\prime }\left(2\pi \right),\;{\mathrm{G̃}}^{\prime \prime }\equiv G^{\prime \prime }/G^{\prime }\left(2\pi \right)$$

For the startup tests, instead, we ran two consecutive measurements on the same specimen: before and after the addition of TCEP. In practice, we first ran a startup test on the raw mucus (*C*_TCEP_ = 0). At the end of the test, the mucus was removed, weighed, and mixed with a TCEP solution at different concentrations (see section 2.2). The sample was then kept at 37 °C for 30 min and finally loaded under the rheometer plates to be retested. The stress curves were normalized according to the following equation:

2 $$\mathrm{\sigma ̃}\left(\gamma ,C_{\mathrm{TCEP}}\right)\equiv \sigma \left(\gamma ,C_{\mathrm{TCEP}}\right)/\sigma \left(\gamma =0.5,C_{\mathrm{TCEP}}=0\right)$$

All tests conducted before the addition of TCEP were used to obtain the average value at *C*_TCEP_ = 0 reported in Figure 1b.

**Figure 1:**
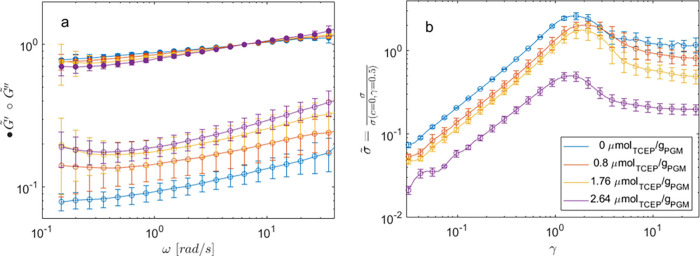
(a) Normalized storage (filled circles) and loss (empty circles) modulus versus frequency ω at different TCEP concentrations (see the legend in panel b). Data are obtained by averaging several frequency sweep tests in the linear regime. (b) Normalized stress versus strain during startup tests at different TCEP concentrations. Error bars represent the standard deviation.

We also estimated the value of the molecular weight between cross-links (*M*_c_) from knowledge of the elastic moduli [*G*′(2π)] at different TCEP concentrations. From the Flory theory,^34^ we have

3 $$M_{c}=RT\rho /G^{\prime }\left(2\pi \right)$$

where *R* is the gas constant, *T* the absolute temperature, and ρ the density of the material (assumed to be 10^9^ g/m^3^). It is worth noting that eq 3 is strictly valid only for homogeneous materials.

### 2.4 Dynamic Light Scattering (DLS)

DLS measurements were conducted with a homemade instrument. The sample was placed into a customized thermostatic cell, consisting of a 10 mm square cuvette. A single-frequency laser beam (model, MLD = 405 nm; Cobolt AB, Solna, Sweden) was focused to a small spot inside the sample. The scattered light was detected by an MDP avalanche photodiode (model PD-050-C0C-FC; Micro Photon Devices srl, Bolzano, Italy) at a scattering angle of 90° to minimize laser flare. Fluctuations in the scattered light intensity over time, as well as the average scattered intensity, were collected and analyzed by an ALV-7004 digital multiple tau correlator (model A7004-016; ALV-GmbH, Lagen, Germany). Particle size distributions were obtained by fitting the intensity correlation function (ICF) with the CONTIN 2DP routine, implemented in the *ALV* software. For each test, the intensity of the laser was adjusted to give a detected count rate on the order of 10 kHz, approximately. The value of the laser power was then used to normalize the scattering intensity. In all measurements, the duration was fixed to 300 s.

To prepare for DLS measurements, raw and reduced mucus samples were first dissolved into PBS at a weight ratio of 1:10 (see the buffer composition in section 2.2), stirred for 15 min, and then centrifuged at 9000 rpm for 15 min. The supernatants were finally collected and analyzed, while the residues were weighed to evaluate the insoluble fractions (see the for details).

### 2.5 Photon Correlation Imaging (PCI)

The PCI technique allows one to measure the dynamics at different points in the sample, combining DLS and imaging.^35^ Measurements were carried out using a homemade setup, as described in Philippe et al.^36^ Briefly, a 405 nm laser beam (provided by Cobolt srl) was collimated to a diameter 1/e^2^ of 4 mm and directed onto the sample. The sample contained in a 0.085-mm-thick cell was supported by a thermostatically controlled metal stand, fixed at *T* = 25 °C. An aspherical lens of focal length *f* = 32 mm was used to image the illuminated sample onto the camera, with a magnification of *M* = 4. An opaque screen with an annular aperture of mean radius *r*_a_ = 4.5 mm and width d*r*_a_ ≈ 0.3 mm was placed in the focal plane of the lens, so that the image was formed only by the scattered light at a scattering vector *q*_PCI_ = 0.97 μm^–1^. Some correspondence was thus established between the points in the sample and the pixels on the camera, allowing measurement of the local dynamics, that is, dynamics on a length scale ξ ≈ π*q*_PCI_^–1^ = 3.24 μm. From the collected images, it was possible to evaluate the correlation function *c*_I_(τ) in space **r** and waiting time *t*_w_:

4 $$c_{I}\left(t_{w},\tau ;r\right)=\frac{{\left\langle I_{p}\left(t_{w}\right)\;I_{p}\left(t_{w}+\tau \right)\right\rangle }_{\mathrm{ROI}\left(r\right)}}{{\left\langle I_{p}\left(t_{w}\right)\right\rangle }_{\mathrm{ROI}\left(r\right)}{\left\langle I_{p}\left(t_{w}+\tau \right)\right\rangle }_{\mathrm{ROI}\left(r\right)}}-1$$

In the equation above, *I*_p_ represents the intensity of the pth pixel, and ⟨···⟩_ROI(*r*)_ denotes an average calculated over a ROI of pixels centered on the position *r*. Specifically, the camera is divided into 48 × 40 ROIs each of 754 pixels. The two-time ICF was evaluated on each pixel and then averaged among the pixels belonging to each ROI.

We also evaluated the mean two-time ICF, *c*_I_(*t*_w_,τ), and the usually reported autocorrelation function, *g*_2_(τ) – 1, by averaging *c*_I_ first over all ROIs and then over *t*_w_, in the case of stationary dynamics: *c*_I_(*t*_w_,τ) ≡ ⟨*c*_I_(*t*_w_,τ;**r**)⟩_*r*_, *g*_2_(τ) – 1 ≡ ⟨*c*_I_(*t*_w_,τ)⟩_*t*_w__.

*c*_I_(τ,*t*_w_) – 1 curves were fitted, at each waiting time *t*_w_, with a generalized correlation function:

5 $$g_{2}\left(\tau ,t_{w}\right)-1=\exp\left[-{\left(\frac{\tau }{\tau_{0}\left(t_{w}\right)}\right)}^{\beta \left(t_{w}\right)}\right]$$

where both parameters (i.e., the β exponent and the decorrelation time τ_0_) depend on *t*_w_.

Finally, to compare the dynamic activity *c*_I_(*t*_w_,τ;**r**), at a fixed decay time τ*, in different regions of the sample, spaced by Δ*r*, we also calculated a “4-point” correlation function *G*_4_(τ*,Δ*r*) by

6 $$G_{4}\left(\tau^{*},\Delta r\right)\equiv {\left\langle \frac{{\left\langle \delta c_{I}\left(t_{w},\tau^{*};r_{1}\right)\;\delta c_{I}\left(t_{w},{\tau^{*};r}_{2}\right)\right\rangle }_{t_{w}}}{\sigma \left(\tau^{*};r_{1}\right)\;\sigma \left({\tau^{*};r}_{2}\right)}\right\rangle }_{\Delta r=\left|r_{1}-r_{2}\right|}$$

Here τ* is a fixed decay time, δ*c*_I_(*t*_w_,τ*;*r*) ≡ *c*_I_(*t*_w_,τ*;*r*) – ⟨*c*_I_(*t*_w_,τ*;*r*)⟩_*t*_w__ represents the local fluctuation of the correlation *c*_I_ at a fixed τ*, and $\sigma \left(\tau^{*};r\right)\equiv \sqrt{{\left\langle {\delta c_{I}\left(t_{w},\tau^{*};r\right)}^{2}\right\rangle }_{t_{w}}}$ is its standard deviation. It is worth noting that, in evaluating *G*_4_, we implicitly assumed that the dynamic properties are stationary.

## 3 Results and Discussion

To assess the effect of disulfide bridge reduction, we started by evaluating the rheological properties of the mucus in the linear and nonlinear viscoelastic regimes. Following the procedure outlined in section 2.3, we report in Figure 1a the normalized average moduli (eq 1), along with their standard deviation, for the linear frequency sweep tests. The mean values of the unnormalized moduli are reported for completeness in Figure S1. As expected,^18,19^ the results show that, by increasing the reducing agent, the mucus undergoes a gradual loss of its solid-like characteristics, as evidenced in Figure 1a by the tendency of ${\mathrm{G̃}}^{\prime \prime }$ to approach ${\mathrm{G̃}}^{\prime }$ . However, the frequency dependence of both moduli shows only minor variation with TCEP. Overall, the soft-gel characteristics of the mucus, i.e., the solid-like character (*G*′ > *G*″) and the weak variation of both moduli with the frequency (∂*G*′/∂ω, and ∂*G*″/∂ω both small), are maintained even at the highest investigated TCEP concentration. The most prominent effect of TCEP on the linear viscoelasticity is the drop, by a factor of about 4.5, of the storage moduli used to normalize the data in Figure 1a [*G* ^′^(2π)]. Their average values at different TCEP concentrations, along with their standard deviations, are reported in Figure 2. We used these values to estimate the molecular weight of the elastic strands (*M*_c_) through eq 3. We found that *M*_c_ ranges from 19 to 87 MDa (Table T2), in agreement with the value reported in the literature for the gel-forming multimer MUC5AC (∼20 MDa).^12,37^ We warn the reader that the obtained *M*_c_ values are valid only for homogeneous samples.

**Figure 2:**
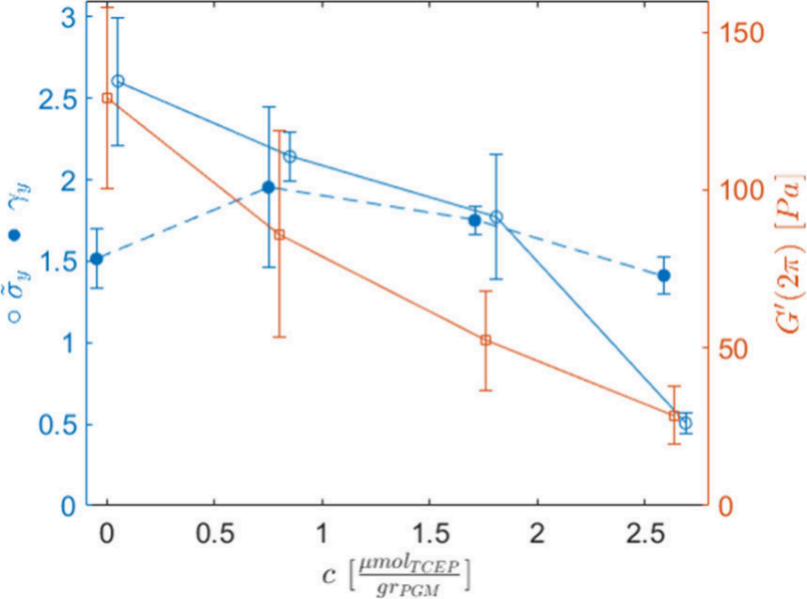
(left axis) Stress and strain evaluated at the yield point and (right axis) average value of the storage modulus at 1 Hz [*G*′(2π)], used in Figure 1a to normalize the moduli, as a function of the TCEP concentration. The yield point data have been shifted horizontally to avoid overlapping error bars.

Moving to the nonlinear case, we evaluated the effect of TCEP on the shear yield point. We then performed startup testing at $\mathrm{\gamma ̇}=1s^{-1}$ by stretching the mucus beyond the limits of its elastic response. In Figure 1b, we report the curves of normalized stress (see eq 2 for normalization) versus strain, averaged on the replicates of samples at fixed TCEP. With increasing γ, the PGM spanning network is first elastically stretched and then passes through a maximum before decreasing toward its steady-state value. We used this overshoot, observed in both viscoelastic and thixotropic materials, to evaluate the yield stress. According to the literature, we referred to it as the “static” yield stress. Its presence, at all investigated TCEP concentrations, is a further indication of the qualitative invariance of the thixotropic nature of the mucus.

We summarize the rheological results in Figure 2, where we report, as a function of TCEP, both the stress and strain at the static yield points, together with the storage modulus used to normalize the data in Figure 1a. Consistent with the linear result, we observed a drop of the static yield stress ( ${\mathrm{\sigma ̃}}_{y}$ ) comparable to the unnormalized storage modulus [*G*′(2π)], while the yield strain remained nearly the same. To confirm these trends, we also estimated the yield point from the amplitude–sweep data at 0.5 Hz, using the tangent method. The data are reported in Table T3. Although these values are different due to the different estimation methods used, the trends are the same as those in Figure 2. From a macroscopic point of view, we can then state that the effect of the reducing agent is only to decrease the degree of cross-linking of the native mucus, which therefore appears “resilient” to TCEP.

At this point, we recall that the data in Figures 1 and 2 are obtained using a gap ranging between 430 and 660 μm (see section 2.3 for details). We apply these gaps to ensure stable measurements at all concentrations. In fact, tests performed at higher gaps for TCEP values greater than 1.76 show the presence of an “instability”. This instability is signaled by a variation of the intrinsic elastoviscoplastic properties (*G*′, *G*″, and σ) as the gap varies. Specifically, tests performed on samples treated with TCEP concentrations of 1.76 and 2.64 μmol_TCEP_/g_PGM_ confirm that a wall slip is at play for gaps greater than ∼700 μm. In Figure 3, we report the evidence of such an effect for the sample at *C*_TCEP_ = 2.64 and, for completeness, for the one at *C*_TCEP_ = 0, where no wall slip is detected. For *C*_TCEP_ = 0, both tan­(δ) and the unnormalized storage modulus *G*′ are independent of the gap, while for *C*_TCEP_ = 2.64, we see an increase in tan­(δ) and a decrease in *G*′ as the gap increases above ∼700 μm.

**Figure 3:**
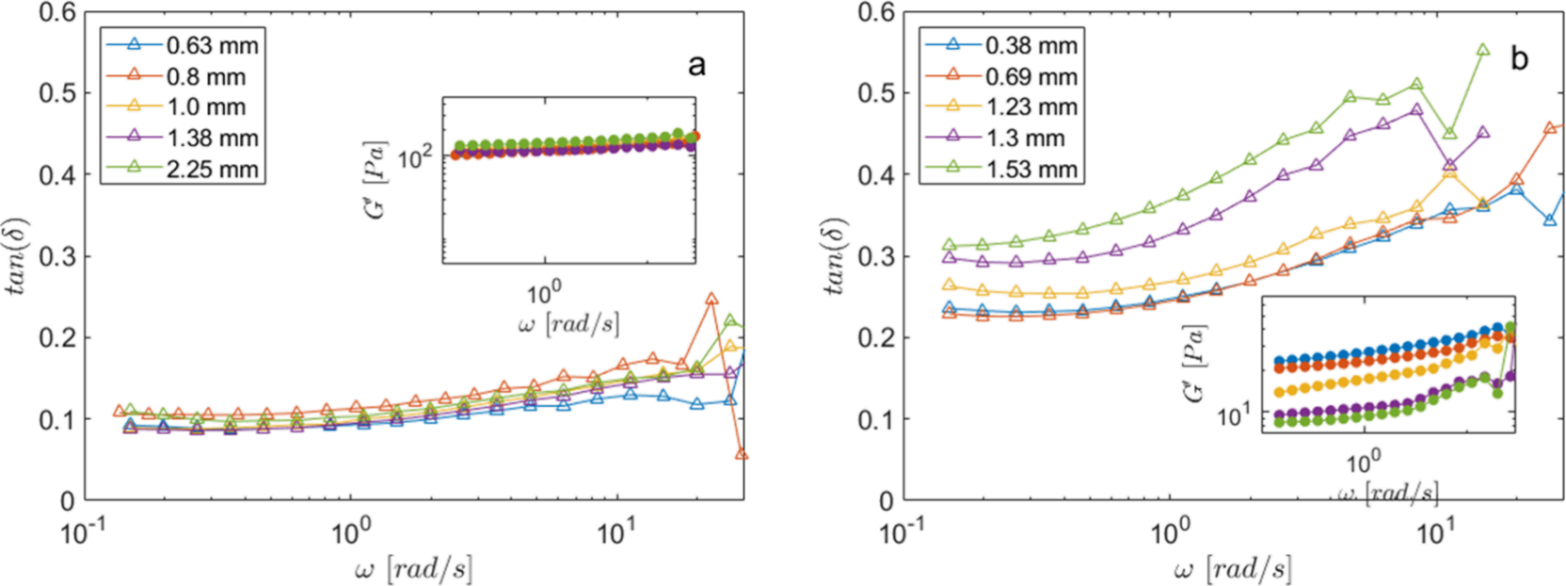
Frequency sweep tests at different gaps, as reported in the legend. As a function of the frequency, we report in the main panel tan­(δ) and in the inset the storage modulus *G*′ for (a) native mucus and (b) mucus treated with 2.64 μmol_TCEP_/g_PGM_.

The presence of a wall slip is known to be related to a lubricating layer near the wall of the rheometer plate. The existence of this layer, only for samples treated at higher TCEP concentrations and only for gaps above a certain threshold, leads to the hypothesis that at such concentrations the mucus is no longer macroscopically homogeneous. As TCEP increases, the mucus is most likely made up of cross-linked gel domains interspersed with fluid ones. That is to say, the mucus becomes more heterogeneous, on a scale on the order of hundreds of micrometers, as the reducing agent increases.

To shed light on the macroheterogeneities induced by the reducing agent, we measured the mucus microscopic dynamics by PCI tests. In Figure 4a–d, we show the mean two-time ICF, *c*_I_(τ,*t*_w_), (see section 2.5) for samples treated with the same four TCEP concentrations previously examined.

**Figure 4:**
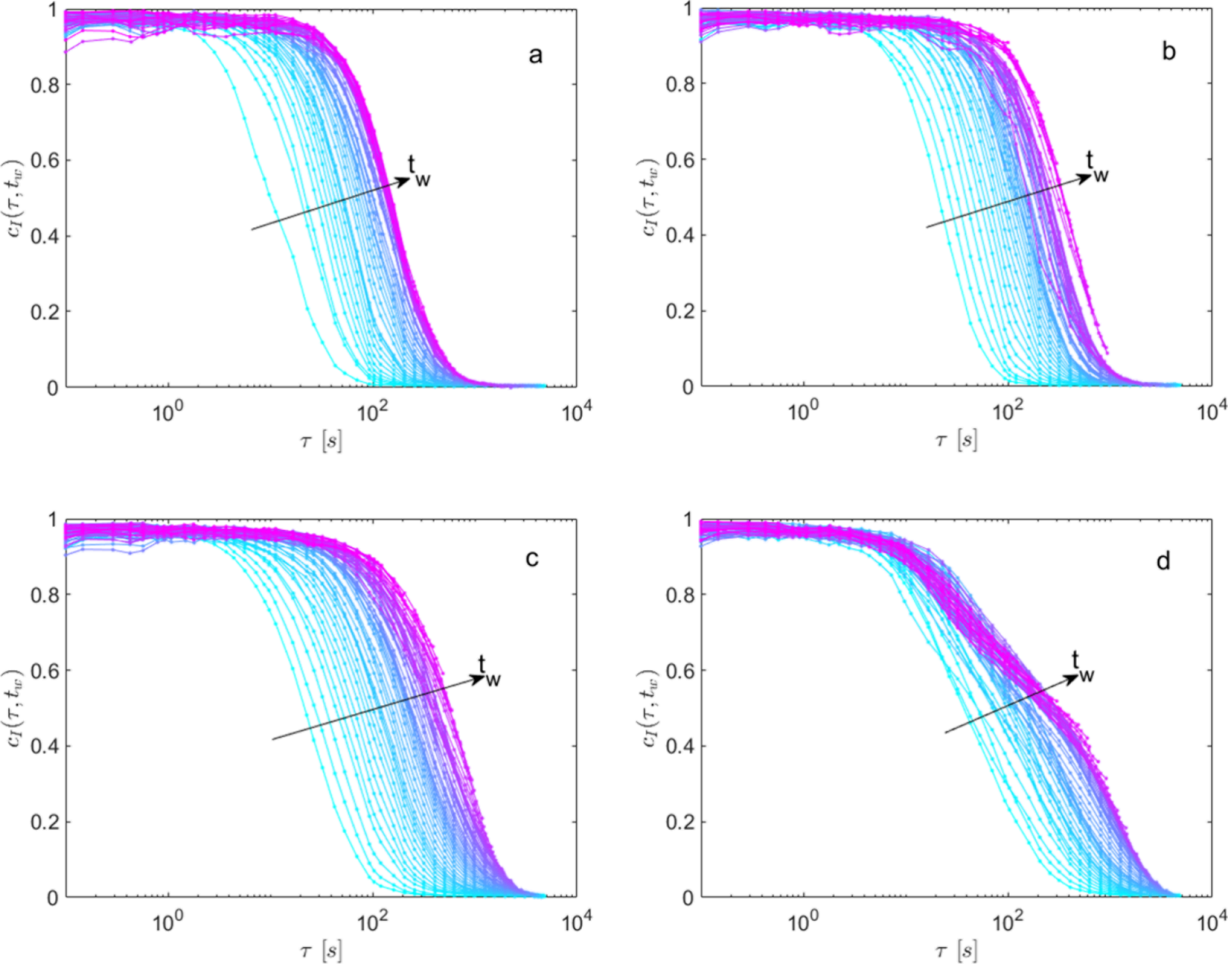
Mean two-time intensity correlation function *c*_I_(τ,*t*_w_) versus decay time τ at different waiting times *t*_w_ and for *q*_PCI_ = 0.97 μm^–1^. The different panels refer to the different TCEP concentrations *C*_TCEP_ (μmol_TCEP_/g_PGM_): (a) 0; (b) 0.8; (c) 1.76; (d) 2.64.

For all TCEP concentrations, the dynamics reported in Figure 4 show a variation with the waiting time (*t*_w_); specifically, as *t*_w_ increases, the correlation curves shift toward increasingly higher values of τ. We quantify these changes by fitting the curves with a generalized exponential decay (eq 5). The main panel of Figure 5 reports the fitting results in terms of the characteristic decorrelation times (τ_0_) as a function of *t*_w_. For the highest investigated TCEP concentration, two characteristic times are actually obtained: the first (magenta full circles) follows the trend observed in the data at lower TCEP; the second (magenta empty circles), instead, remains almost constant throughout the experimental window. For those characteristic times displaying a change with *t*_w_ (filled symbols in Figure 5), we observe a common feature: as *t*_w_ increases, the rates of τ_0_ reduce until reaching a pseudoplateau value, characterized by increasingly higher values of τ_0_ with TCEP.

**Figure 5:**
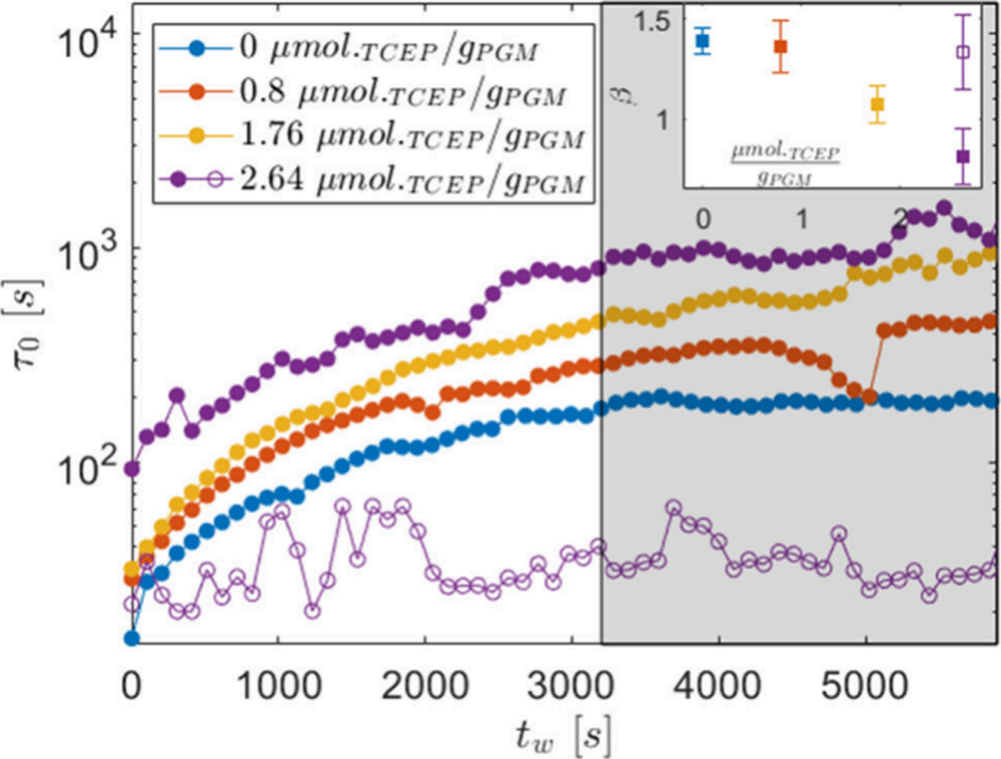
Main: Evolution of the characteristic decay time τ_0_(*t*_w_) for the four concentrations studied (see the legend), obtained by fitting the data in Figure 4 with a single (double for *C*_TCEP_ = 2.64) generalized exponential function. Inset: Average exponential factor β, for *t*_w_ ≳ 3200 s, versus TCEP concentration.

A behavior like the one displayed in Figures 4 and 5 was already observed for combined Rheo-DLS experiments conducted on untreated PGM (i.e., as for the data in Figure 4a).^32^ In that case, it was found that a modest imposed shear stress is responsible for an enhancement in mucus reorganization, which was detected by a transient acceleration of the microscopic dynamics. Consistently, in the case at hand, we attribute the observed dynamics to stress induced during the procedure of sample loading inside the optical cell. Following loading, in fact, the sample undergoes an unavoidable (and somehow uncontrolled) uniaxial compression between the thin slit of the cell windows, which causes the observed variation in *c*_I_(τ,*t*_w_). As time goes on, the imposed stress relaxes, and the dynamics slow down until reaching a pseudoplateau value (ideal sample at rest). The dynamics measured at rest, i.e., for *t*_w_ ≳ 3200 s, in our case, are therefore affected only by the residual stress inside the mucus. The average values of the characteristic times $\tau_{\infty }\equiv {\left\langle \tau_{0}\left(t_{w}\right)\right\rangle }_{t_{w}>3200}$ are thus indicative of these residual stresses: the longer the decay time, the lower the stress in the gel.^38^ We can therefore say that, as the TCEP increases, *c*_I_(τ,*t*_w_) displays a smaller and smaller residual stress, consistent with the rheological variations observed previously.

In parallel to τ_∞_, we also calculate the average value of the exponent β: a quantity indicative of the diffusion mechanism inside the sample^38^ (see the inset in Figure 5). Clearly, in the limiting case of *C*_TCEP_ = 2.64, we report both values of β, associated with the two τ_∞_. Overall, the β values are lower than 2 and decrease with TCEP for those dynamics changing with *t*_w_ (filled symbols in Figure 5). We recall that a value of β < 2 is indicative of a broad distribution of ballistic dynamics. As reported in ref 38, for β < 2, the distribution of the scatterer’s velocity (*v*) follows a power law: *P*(*v*) ∝ *v* ^–(β+1)^. Hence, the lower β, the wider the distribution of those velocities. The trend of β versus *C*_TCEP_ then allows us to infer that the dynamics at pseudosteady state are characterized by a distribution of decay times that become increasingly broader with the reducing agent. In particular, at *C*_TCEP_ = 2.64, such a distribution ends up assuming a bimodal form: with a small time associated with a “high” residual stress (magenta empty square in the inset of Figure 5) and a larger time associated, instead, with a “low” residual stress (magenta filled square). The results in Figures 4 and 5 then support the idea outlined based on the instabilities in the rheological data, according to which mucus becomes more heterogeneous as the reducing agent increases.

To further confirm this picture, we exploit the features of the PCI method by resolving, this time, the mucus dynamics in both space and times *c*_I_(τ,*t*_w_;*r*). Once again, we focus on times *t*_w_ ≳ 3200 s. For the pseudosteady state, we then construct a dynamic activity map (DAM), by evaluating the decay time (τ_06_), i.e., the time for which *c*_I_ – 1 = 0.6, as a function of *t*_w_ at different positions *r* inside the mucus.^35^ Figure 6 reports a snapshot of these DAMs at the different TCEP concentrations (DAM movies can be found in the Supporting Information). The DAMs clearly show the presence within the illuminated sample of regions with different dynamics. The increase in the distribution of decay times, as revealed in the inset of Figure 5, can now be recognized as stemming from the presence of domains with different microscopic dynamics. The DAMs then unveil an increasing spatial fluctuation with an increasing TCEP concentration.

**Figure 6:**
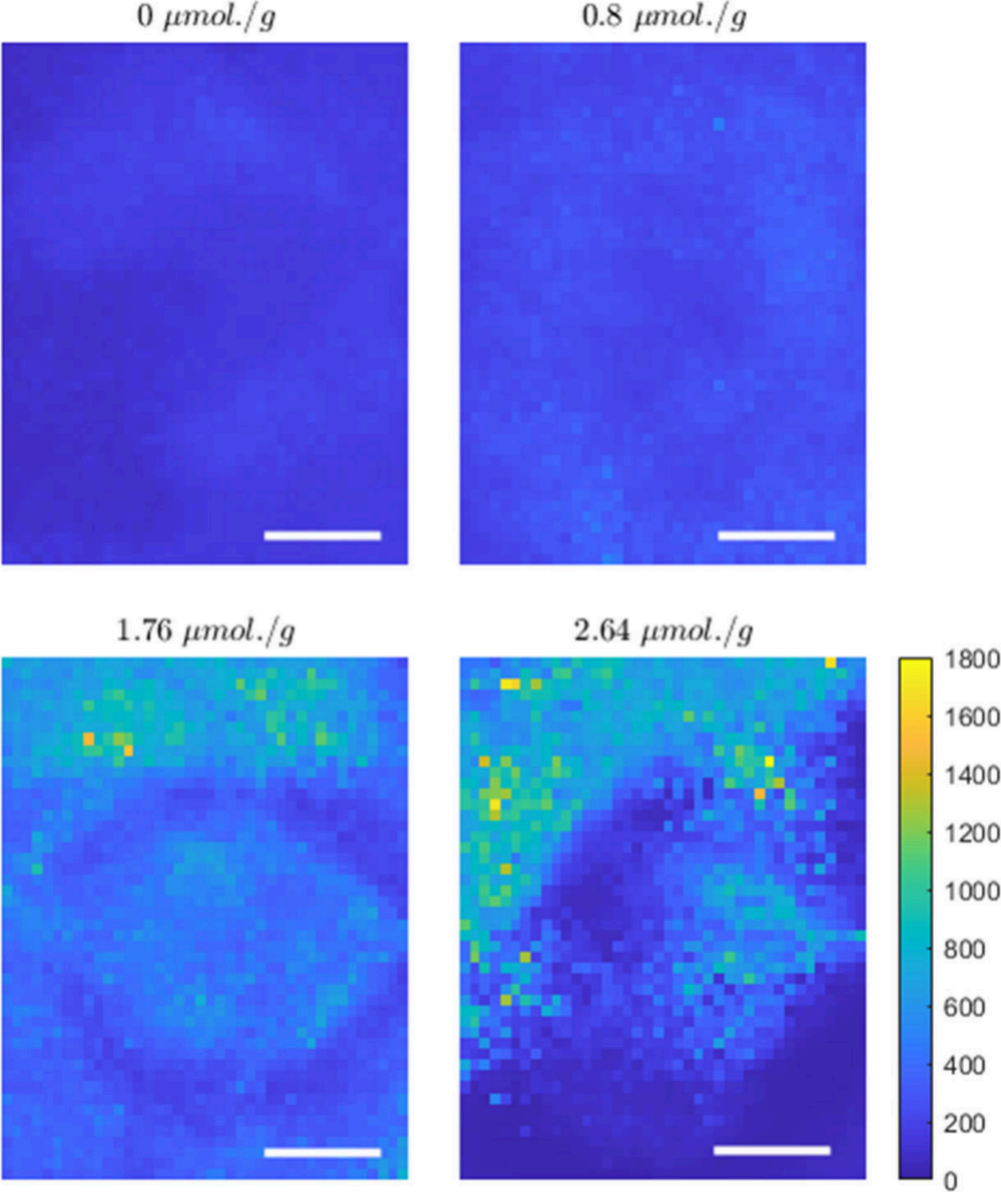
Snapshots of the dynamic activity maps for the four TCEP concentrations investigated, showing the local decorrelation time for *c*_I_(τ,*t*_w_;*r*) = 0.6 during the pseudosteady state of PCI tests (*t*_w_ ≳ 3200 s). Scale bars correspond to 500 μm.

To quantify these fluctuations, we also calculate the four-point correlation function *G*_4_(Δ*r*,τ) (see eq 6) and display the results in Figure 7. Due to the noise on the individual ROIs, we only report the values for Δ*r* > 0, having rescaled *G*_4_ so that it goes to 1 when Δ*r* goes to 0.^35^

**Figure 7:**
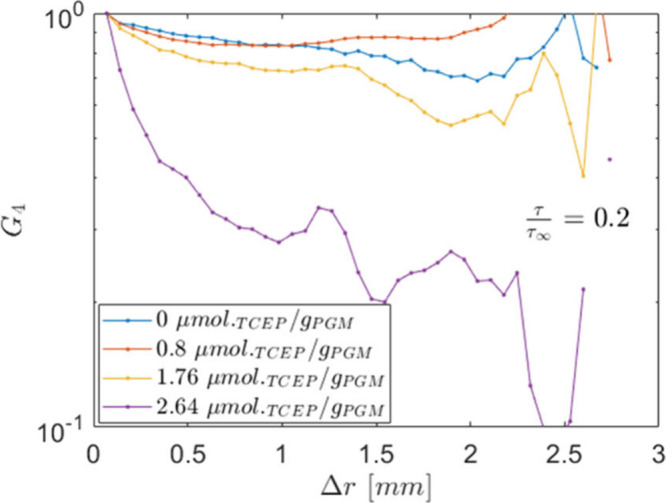
Spatial correlation of the dynamics evaluated from eq 6 for τ/τ_∞_ = 0.2, during the pseudosteady state regime (*t*_w_ ≳ 3200 s).

The trends in Figure 7 show that samples at concentrations lower than *C*_TCEP_ < 2.64 are characterized by a long-range dynamic correlation that extends for about 1 mm before decreasing. On the contrary, the sample treated with the highest TCEP concentration displays an immediate decay, followed by a more gradual decrease, again at Δ*r* around 1 mm. The presence of long-range spatial correlations is quite common in gels and originated by a “strong” connectivity of the gel strands inside the macroscopic material domains. On a macroscale, the behavior displayed in Figure 7 is then a signature of a “strong” interconnected gel for *C*_TCEP_ < 2.64, which becomes “weaker” starting from *C*_TCEP_ = 2.64.

Last, we assess the selectivity, at the molecular level, of TCEP on different disulfide bonds. We refer here to the specificity of thiol reducing agents in reacting with the different types of disulfide bonds present in the native mucus: namely, mucin intrachain bonds, mucin interchain bonds, and bonds on nonmucin components. A different selectivity can, in fact, give rise to two different interpretations of the mechanism leading to the viscoelasticity drop: (i) a lowering of the mucins’ molecular weight, as supposed so far by most of the literature;^18,22,23^ (ii) a change in the molecular conformation, with a consequent reduction of gel connectivities, as suggested recently.^21^ Following the protocol reported in section 2.4, we suspended the various treated mucus samples in PBS and analyzed the soluble fraction (supernatant) through a traditional DLS technique. In Figure 8, we report the average ICF versus τ, and the normalized scattered intensity as a function of TCEP.

**Figure 8:**
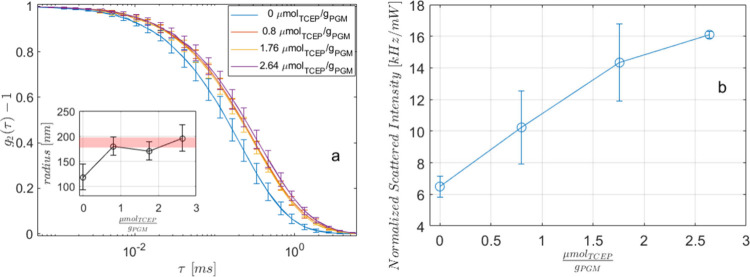
(a) Main: Intensity autocorrelation functions versus decay time for solubilized mucus after treatment with different TCEP concentrations (as in the legend). Inset: Mean radius as a function of the TCEP concentration. The pink shadow region is the range of radii measured for unreduced MUC5AC reported in ref 12. (b) Normalized scattered intensity versus TCEP concentration (see section 2.4). Error bars represent the standard deviation among the different replications.

Except for the data at *C*_TCEP_ = 0, the decorrelation curves are superimposable, indicating an invariance in the characteristic size of suspended macromolecules. Their mean radii, obtained from the CONTIN analysis, are plotted in the inset of Figure 8a and are consistent with the size range reported by other authors on gel-forming mucins MUC5AC (pink shadow).^12,37^ Contrary to the constant value shown by the radius, the intensity of the scattered light (Figure 8b) increases almost linearly with increasing TCEP, confirming, where necessary, the copresence of an ever-increasing soluble fraction within the reduced mucus gels. For completeness, we report the data of the insoluble fractions in Table T4. As expected, the insoluble fraction reduces with TCEP, consistent with the data in Figure 8b. Regarding the lower value of the mean radius at *C*_TCEP_ = 0, along with its lower soluble fraction, it is plausible to hypothesize that this is a consequence of the strength of the unreduced gel-forming mucins to remain adherent to the gel phase. In other words, in native mucus, the fraction dissolved by PBS lacks high-molecular-weight mucins.

On the basis of the results of Figure 8, we infer that the second hypothesized mechanism is the one at play in our system. We suggest, in line with the literature, that disulfide bonds of nonmucin components (e.g., TFFs) and intramucin ones are the main TCEP’s target sites. Upon reduction, these macromolecules undergo a conformational change that leads to the observed experimental results. It is worth noting that these TFFs are more abundant in gastric and intestinal mucus, which could explain the contradictory results reported in the literature. A word of caution is in order here because measurements conducted with DLS tend to be biased toward high molecular weights. This effect occurs as a consequence of the dependence of the scattered intensity to the scatterer radius: *I*_scatt_ ∝ *r* ^6^. Consequently, smaller objects contribute less to the ICF.

From the molecular prospective, we hypothesize that the mechanism leading to the observed experimental evidence is related to the spontaneous tendency of mucins to phase separate, as reported in ref 36. This tendency is accelerated by the reduction of the degree of cross-linking and leads to an increasingly heterogeneous distribution of macromolecules as TCEP increases. Such heterogeneities might then give rise to regions that behave similarly to the unreduced sample, surrounded by more fluid domains. In simple terms, the stickiness of the mucus components would reorganize it into macrodomains following the cleavage of disulfide bonds. In this scenario, the result shown by the rheological tests conducted at gaps comparable to the size of these macrodomains should be attributed to the viscoelasticity of these domains. The expected spectra would therefore be similar to those of native mucus (*C*_TCEP_ = 0), although shifted proportionally to the fraction of macrodomains.

In Figure 9, we schematically illustrate our hypothesis on the mechanism of action, at the molecular scale, of TCEP on PGM. Under native conditions (Figure 9a), PGM appears to be homogeneous on the mesoscopic scale, with a size of strands between cross-links that compares to the molecular weight of the most abundant gel-forming component (MUC5AC). In line with the prevailing literature,^27^ we assume that hydrophobic interactions (shown as blue dots) are cross-linking the PGM macromolecules (shown as black coils), conferring the elastic modulus its nearly flat mechanical spectrum. Upon disulfide bond reduction (Figure 9b), we macroscopically observe a drop in the viscoelastic properties, which, according to DLS measurements, does not, however, alter the mucins’ molecular weight. In line with the recent literature,^21^ we assume that TCEP mainly reduces intramucin and nonmucin disulfide bonds. In turn, this reduction causes an unfolding of the mucin and nonmucin macromolecules (shown now in green), with consequent exposure of hydrophobic amino acidic units that were previously hidden.^3,39^ At the macroscopic level, these effects translate into a destabilized network, with concomitant acceleration toward a phase-separated mucus.

**Figure 9:**
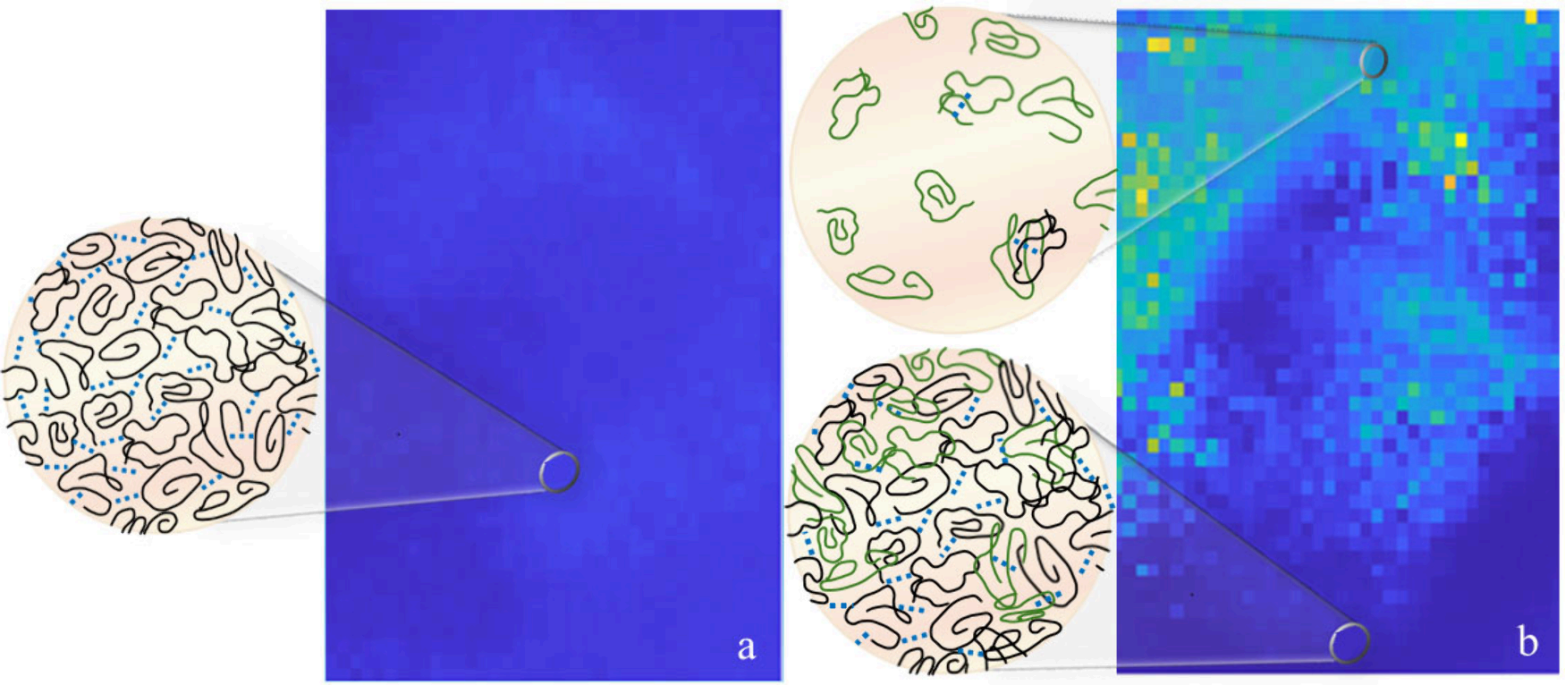
Conceptual model of the chemical mechanism by which TCEP acts on PGM macromolecules (here represented as random coils), leading to an alteration of the viscoelastic properties. In native mucus (a), the gel network appears homogeneous on a mesoscopic scale, with hydrophobic interactions (shown by blue dots) that give mucus its solid characteristic. After the addition of TCEP (b), we have a change in the conformation of the macromers (schematically described by a change in the color of some of the coils) with a reorganization of the mucin network, leading to a phase-separated system.

## 4 Conclusion

We reported evidence of the formation of macroheterogeneity, on the order of hundreds of microns, within native PGM as the concentration of the disulfide reducing agent increases. Indeed, for the TCEP concentrations studied here, the mucus undergoes a partial disaggregation together with an increase in its heterogeneity. This macroheterogeneity was detected both through rheological measurements at different gaps and through measurements on spatially resolved microscopic dynamics. It is worth noticing that the mentioned “resilience” of mucus to TCEP, i.e., the persistence of soft-gel characteristic observed in rheological tests, can be explained as a mere consequence of such macroheterogeneities. Indeed, considering the rheological response of the macroseparated system as the sum of contributions from the different domains, we expect the reduced samples to be dominated by the fraction with the highest viscoelastic properties (i.e., the more cross-linked one).

As a further outcome, we also indicated that TCEP does not alter the mucin molecular weight of native PGM. Our data support the hypothesis that reducing agents act on mucin and nonmucin proteins (TFFs) by solely inducing a change of their conformation, which, in turn, reduces mucus connectivity.

Finally, we emphasize that, although there are nonmarginal differences in gastric versus other mucus sources, we speculate that the macroscopic behavior exhibited here could be found also on other tissue sources. This consideration arises from the idea that the macroscopic biophysical behavior of mucus is intimately connected to the stickiness of the mucus components and, therefore, common to many tissue sources. Consequently, the macroheterogeneity observed here could be expected also for lung mucus, where we hypothesize that it could be helpful in examining the phenomena of mucus removal by coughing. In this sense, a macroheterogeneous disaggregation of mucus would decrease local resistance, facilitating expectoration.

## Supplementary Material











## Notes

Gaps used in the startup test, rheological tests, raw phase angle versus frequency, molecular weight of the elastic strand, yield stress point, and insoluble fractions (PDF)
DAM for the native PGM sample (*C*_TCEP_ = 0) (AVI)
DAM for the PGM sample treated with 0.8 μmol_TCEP_/g_PGM_ (AVI)
DAM for the PGM sample treated with 1.76 μmol_TCEP_/g_PGM_ (AVI)
DAM for the PGM sample treated with 2.64 μmol_TCEP_/g_PGM_ (AVI)

## Abbreviations

DAM: dynamic activity map
ICF: intensity correlation function
MCC: mucociliary clearance
PBS: phosphate buffer solution
PCI: photon correlation imaging
PGM: porcine gastric mucus
TCEP: tris­(2-carboxyethyl)­phosphine hydrochloride
TFFs: trefoil factors
