# Supplementary material for: Macroheterogeneities Induced by Disulfide Bond Reduction in Native Mucus
Source: ACS Appl Bio Mater. 2025 May 19;8(6):4647–56. doi: 10.1021/acsabm.4c01758 (PMC12175130; doi:10.1021/acsabm.4c01758)
Supplement: Supplementary file 1 [file mt4c01758_si_001.pdf]

Supporting Information for:

Macro-heterogeneities induced by disulfide bond reduction in native mucus

*Giorgia Franzino, Fabiana Tescione, and Domenico Larobina**

Institute of Polymers, Composites, and Biomaterials of the National Research Council of Italy, P.le E. Fermi 1, 80055 Portici, Naples, Italy

Corresponding author. Email: domenico.larobina@cnr.it

1. GAPS USED IN THE STARTUP TEST

In table T1 we report all the gaps used for the startup test of figure 1b; that is, before and after the addition of TCEP. We signal that the gaps with the largest values are always related to the unreduced samples ($C_{TCEP} = 0$).

$C_{TCEP} \left[\frac{\mu\text{mol}}{\text{g}_{\text{PGM}}}\right]$	0	0.8	0	1.76	0	2.64
$h\ [mm]$	0.66	0.59	0.60	0.55	0.61	0.46
	0.6	0.53	0.6	0.5	0.64	0.52
	0.51	0.47	0.62	0.55	0.55	0.44

Table T1. Gaps used in the startup tests reported in figure 1b.

2. RHEOLOGICAL TESTS

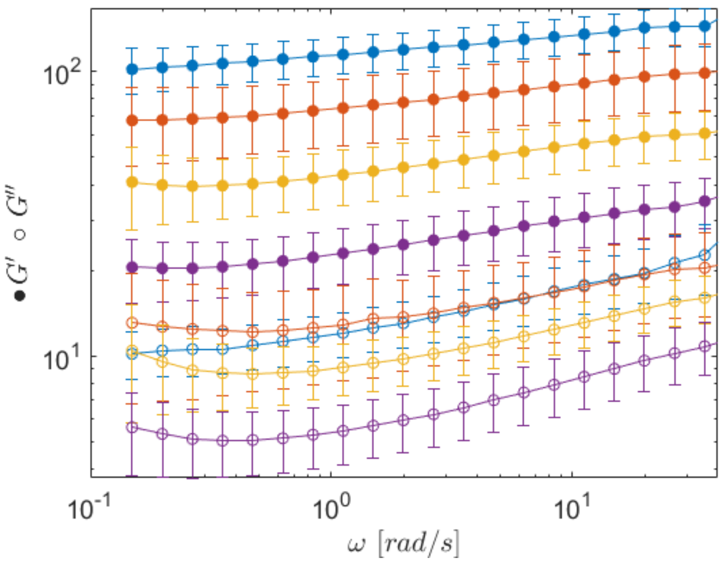

Figure S1 Unnormalized average storage (filled circles) and loss (empty circles) modulus vs. frequency ω at different TCEP concentrations: $C_{TCEP} = 0$ (blu); $C_{TCEP} = 0.8$ (red); $C_{TCEP} = 1.76$ (yellow); $C_{TCEP} = 2.64$ (magenta). Error bars have been reduced by a factor of 1.5 to avoid overlapping.

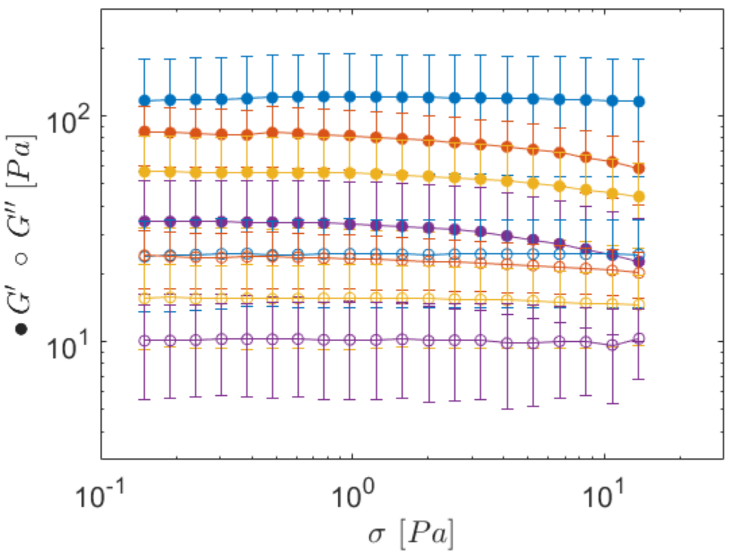

Figure S2 Average shear moduli vs. stress at 5 Hz for the four different TCEP concentrations investigated. Symbols and color code are as in figure S1.

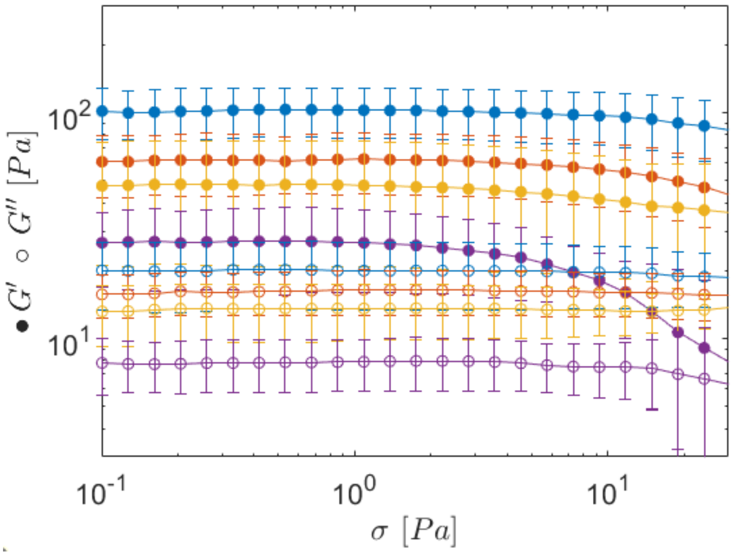

Figure S3 The same as in figure S2 but with f = 0.5 Hz.

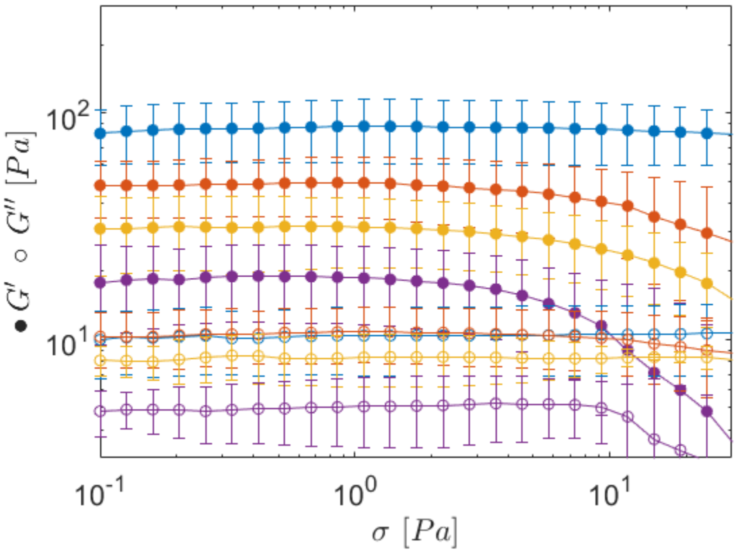

Figure S4 The same as in figure S2 but with f = 0.05 Hz.

3. RAW PHASE ANGLE VS. FREQUENCY

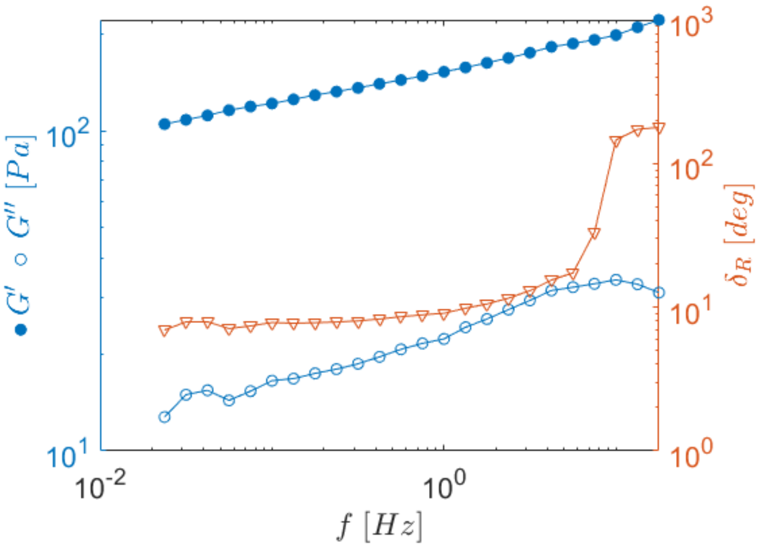

Figure S5 Representative frequency sweep test conducted on native mucus ($C_{TCEP} = 0$), following the procedure reported in section 2.3 of the main text. On the left axis we show the moduli, while on the right axis we show the raw phase angle.

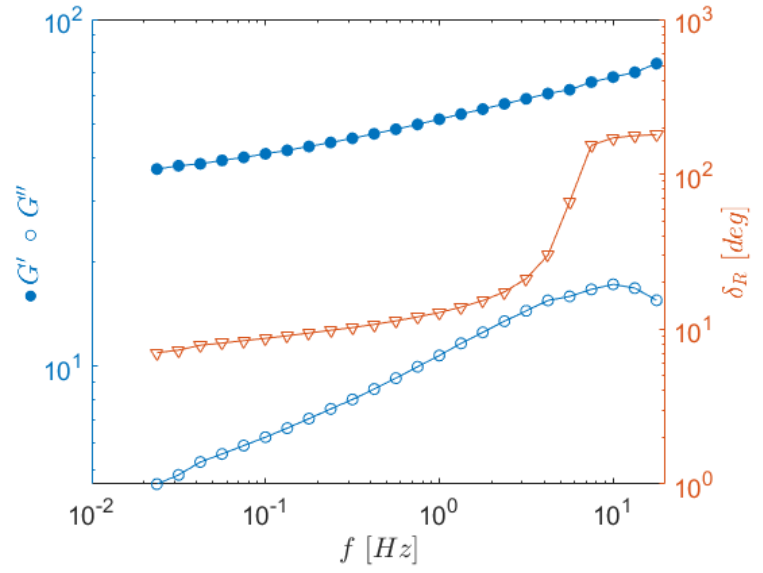

Figure S6. Same as in figure S5 but with $C_{TCEP} = 1.76$.

4. MOLECULAR WEIGHT OF THE ELASTIC STRAND

$C_{TCEP}\ [\frac{\mu mol}{g_{TCEP}}]$	**0**	**0.8**	**1.76**	**2.64**
$M_{w,strand}$ [MDa]	19	29	48	87

Table T2 Estimation of the molecular weight of the mucus gel elastic strand as a function of TCEP concentration. The values are calculated from the 1 Hz elastic modulus via eq.3 reported in the Material and Methods section.

5. YIELD STRESS POINT

For the amplitude-sweep data at 0.5 Hz we evaluate the stress at which elastic modulus G' departure by 10% from its plateau value at low σ. We recall that this stress corresponds to the end of the linear regime and is often used as an alternative method to determine experimentally the yield stress of materials.

$C_{TCEP}\ \left[\frac{\mu mol}{g_{TCEP}}\right]$	*0*	*0.8*	*1.76*	*2.64*
$\sigma_c\ [Pa]$	17.0	10.6	6.63	3.22
γ_c	0.177	0.179	0.153	0.132

Table T3 Yield stress point extracted from amplitude sweep tests at 0.5 Hz by the tangent method.

It is worth noticing that the values obtained by the above method are lower than the one deduced from the maximum in the start-up experiment (see figure 2 in the manuscript). This is expected, since the former are detecting the departure from the linear behavior, while the latter corresponds to the rupture of the gel network. Nevertheless, data in table

T3 are consistent with the one reported in figure 2 of the main text. In both cases, in fact, we observe a decrease in the yield stress as TCEP increases, while the corresponding strains remain approximately constant.

6. INSOLUBLE FRACTIONS

To calculate the insoluble fractions at the four investigated TCEP concentrations we first follow the procedure reported in section 2.4. Briefly, after dissolving the reduced samples we centrifuge and finally separate the supernatants from the residues. Due to the swelling of the mucus samples, the percentage evaluated from the wet residues (w_{wet}), i.e., as the ratio of the collected wet residues over the initial mucus weights, in some cases ($C_{TCEP} = 0$ and 0.8) far exceeds 100%. To circumvent this issue, we dry all residues, along with the undissolved sample (raw mucus), and evaluate the insoluble fraction (w_{dry}) as ratio of the dried residues over the dried undissolved mucus (i.e., the initial solid fraction). In Table T4, we report both the average fractions along with their standard deviation on a triplicate measure.

C_{TCEP} $[\frac{\mu mol}{g_{TCEP}}]$	**0**	**0.8**	**1.76**	**2.64**
w_{wet}	1.50±0.079	1.46±0.718	0.617±0.232	0.348±0.135
w_{dry}	1.06±0.167	0.915±0.155	0.468±0.149	0.344±0.059

Table T4 Insoluble weight fraction of reduced mucus as function of the TCEP concentrations.
